# Novel Plasma Biomarker-Based Model for Predicting Acute Kidney Injury After Cardiac Surgery: A Case Control Study

**DOI:** 10.3389/fmed.2021.799516

**Published:** 2022-01-14

**Authors:** Yichi Zhang, Haige Zhao, Qun Su, Cuili Wang, Hongjun Chen, Lingling Shen, Liang Ma, Tingting Zhu, Wenqing Chen, Hong Jiang, Jianghua Chen

**Affiliations:** ^1^Kidney Disease Center, The First Affiliated Hospital, College of Medicine, Zhejiang University, Hangzhou, China; ^2^Key Laboratory of Nephropathy, Hangzhou, China; ^3^Institute of Nephropathy, Zhejiang University, Hangzhou, China; ^4^Zhejiang Clinical Research Center of Kidney and Urinary System Disease, Hangzhou, China; ^5^Department of Cardiothoracic Surgery, The First Affiliated Hospital, Zhejiang University School of Medicine, Hangzhou, China; ^6^Department of Intensive Care Unit, The First Affiliated Hospital, Zhejiang University, Hangzhou, China

**Keywords:** acute kidney injury, biomarkers, WGCNA, CSA-AKI, predictive model, GDF-15, IL1RL1, uPAR

## Abstract

**Introduction:**

Acute kidney injury (AKI) after cardiac surgery is independently associated with a prolonged hospital stay, increased cost of care, and increased post-operative mortality. Delayed elevation of serum creatinine (SCr) levels requires novel biomarkers to provide a prediction of AKI after cardiac surgery. Our objective was to find a novel blood biomarkers combination to construct a model for predicting AKI after cardiac surgery and risk stratification.

**Methods:**

This was a case-control study. Weighted Gene Co-expression Network Analysis (WGCNA) was applied to Gene Expression Omnibus (GEO) dataset GSE30718 to seek potential biomarkers associated with AKI. We measured biomarker levels in venous blood samples of 67 patients with AKI after cardiac surgery and 59 control patients in two cohorts. Clinical data were collected. We developed a multi-biomarker model for predicting cardiac-surgery-associated AKI and compared it with a traditional clinical-factor-based model.

**Results:**

From bioinformatics analysis and previous articles, we found 6 potential plasma biomarkers for the prediction of AKI. Among them, 3 biomarkers, such as growth differentiation factor 15 (GDF15), soluble suppression of tumorigenicity 2 (ST2, IL1RL1), and soluble urokinase plasminogen activator receptor (uPAR) were found to have prediction ability for AKI (area under the curve [AUC] > 0.6) in patients undergoing cardiac surgery. They were then incorporated into a multi-biomarker model for predicting AKI (C-statistic: 0.84, Brier 0.15) which outperformed the traditional clinical-factor-based model (C-statistic: 0.73, Brier 0.16).

**Conclusion:**

Our research validated a promising plasma multi-biomarker model for predicting AKI after cardiac surgery.

## Introduction

Acute kidney injury (AKI), a common perioperative complication, happens in 5–42% of patients going through adult cardiac surgery. About 2–3% of patients need renal replacement therapy (RRT) after cardiac surgery. Cardiac-associated acute kidney injury (CSA-AKI) is independently associated with a prolonged hospital stay, increased cost of care, and increased post-operative mortality (1–3). Even those who get only transient perioperative AKI at stage 1 or 2 take a significantly increased risk of death as well as a higher risk of chronic kidney disease (4–6).

All consensus guidelines, such as the Kidney Disease: Improving Global Outcomes (KDIGO) Guidelines rely on two essential criteria to define and stage AKI, which are a reduction in urinary output and increased serum creatinine (SCr) level. Using SCr level and urine output presents some challenges for AKI detection. First, the widely used diuretics in the perioperative period make it hard to detect AKI based on oliguria. Second, a rise in SCr level is delayed following renal dysfunction, which makes it a deficient biomarker for the early detection of AKI. The glomerular filtration rate (GFR) of patients can decrease significantly while SCr level shows minimal fluctuation (7). Cardiopulmonary bypass (CPB) and fluid administration cause hemodilution, which allows post-operative SCr level likely to fall below the pre-operative baseline, thus probably delaying the recognition of AKI (8).

Owing to the limitations of SCr and urinary output, much work has been done to identify biomarkers which can offer pre-operative risk stratification and early detection of AKI (9). Risk stratification of AKI helps to provide preventative measures and advises patients about possible bad outcome that patients face after cardiac surgery (7). Early identification along with proper intervention has been reported to decrease the frequency and severity of AKI after cardiac surgery, showing the prospect of early diagnosis (10).

A lot of risk evaluation scores for post-operative AKI based on demographic data were put forward, such as Cleveland Clinic (11), the Metha (12), and the Birnie scores (13), but they were designed to discriminate post-operative AKI that require RRT. Other than the urine indicators, such as neutrophil gelatinase-associated lipocalin (NGAL), kidney injury (KIM1), liver-type fatty-acid-binding protein (L-FABP), insulin-like growth factor-binding protein-7 (IGFBP-7), and tissue inhibitor of metalloproteinase-2 (TIMP2), plasma biomarkers deserve exploration due to their continued participation in the inflammatory response during AKI development and disease prognosis (14).

Cardiac-associated acute kidney injury involves multidimensional networks of molecules and cells. Thus, the sensitivity of individual genes can be low while network analysis is informative, especially when the available expression data is a large set. Weighted Gene Co-expression Network Analysis (WGCNA) is a vital method to perceive the gene function and gene association from genome data (15). WGCNA can be applied to detect co-expression modules of highly correlated genes and target modules associated with clinical traits, which offers information about foreseeing the functions of co-expression genes and discovering genes that have significant roles in target diseases (16).

**Figure 1:**
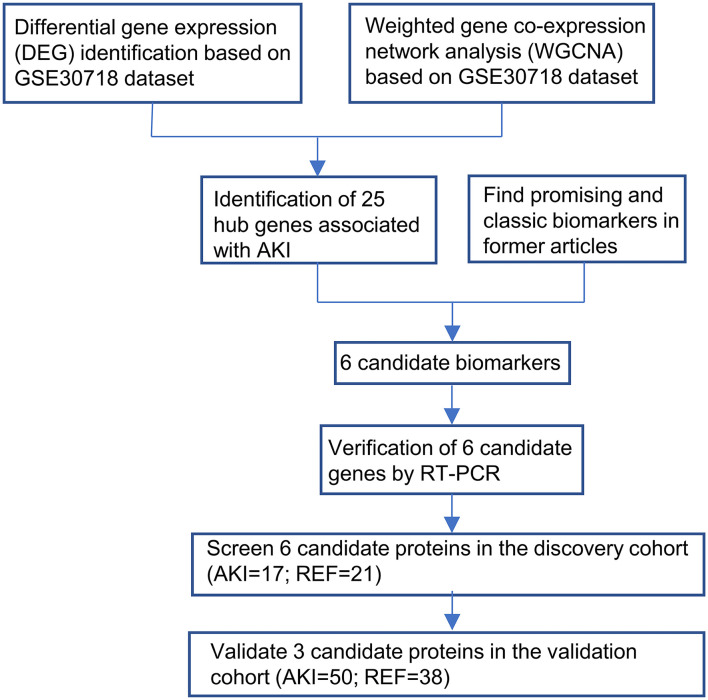
Workflow for the whole study was shown in the flowchart.

In this study, we performed the WGCNA and reviewed previous articles to find 6 potential biomarkers for prediction. Then, we collected peripheral blood mononuclear cells (PBMCs) RNA and plasma of patients undergoing cardiac surgery with or without AKI. The prediction capability of these 6 candidates was evaluated in the discovery cohort and 3 of them came out with better predictive values, which were further verified in the validation cohort. For better clinical practice, a multi-biomarker model for predicting CSA-AKI was then built. The workflow of the study was shown in a flowchart (Figure 1).

## Methods

### Differentially Expressed Gene (DEG) Analysis and WGCNA

The GSE30718 dataset was downloaded from the Gene Expression Omnibus (GEO) database (http://www.ncbi.nlm.nih.gov/geo/) and the platform used was the GPL570 [HG-U133_Plus_2] Affymetrix Human Genome U133 Plus 2.0 Array (Affymetrix, Santa Clara, CA, USA) (17). We determined differentially expressed genes (DEGs) using the Linear Models for Microarray (Limma) package for RNA-seq in the R platform. Genes with |log FC| > 1 and an adjusted *p* < 0.05 were regarded as DEGs. WGCNA package was used to identify AKI-related modules and find hub genes in significant modules. We calculated gene significance (GS), which is the absolute value of the correlation coefficient between genes and outcome, and module membership (MM) of the gene in the module defined as the relation to expression profiles. Hub genes of significant modules were filtered by GS (|GS| > 0.2), MM (|MM| > 0.8), and DEG.

### Patient Selection, Data Collection, and Definitions

Between October 2020 and March 2021, 425 patients undergoing elective cardiac surgery were recruited from the surgical intensive care unit (ICU) of the hospital, which contained 131 patients in the discovery cohort and 294 in the validation cohort. Eligible participants underwent cardiac surgery with CPB in the center. Patients were excluded if they met any of the following criteria: (1) aged <18 or >80 years; (2) history of renal transplantation or dialysis; (3) exposure to nephrotoxic drugs within 2 weeks before surgery; (4) comorbidity of advanced chronic kidney disease; and (5) recent urinary tract infection or obstruction. Nephrotoxic drugs include aminoglycoside antibiotics, glycopeptide antibiotics, antifungal drugs, antiviral drugs, beta-lactam antibiotics, other antimicrobial drugs, diuretics, or dehydration drugs, non-steroidal anti-inflammatory drugs, chemotherapy, and traditional Chinese medicine. All patients were followed until discharge or hospital mortality. Clinical data were collected by reviewing the hospital database.

The diagnosis of AKI was established according to the KDIGO guidelines as follows: AKI stage-1: increase in SCr by ≥0.3 mg/dl (≥26.5 μmol/l) within 48 h or increase in SCr to 1.5~1.9 times baseline within prior 7 days; AKI stage-2: increase in SCr to 2.0~2.9 times baseline; AKI stage-3: increase in SCr to 3 times baseline or an increase in SCr by 354 μmol/L or when the patient initiated RRT. RRT was administered for uremia, volume overload, or biochemical abnormalities, according to institutional protocols. The outcome was AKI.

### Sample Collection and Plasma Biomarker Measurement

Venous blood samples were uniformly collected from all participants into EDTA (Ethylene Diamine Tetraacetic Acid) anticoagulation tubes before an operation, at admission to the ICU (between 0.5 and 1 h after cardiac surgery), morning (6:00 a.m.) of the first post-operative day (between 8 and 18 h after cardiac surgery) and the second post-operative day. At a 4°C temperature, the samples were centrifuged at 1,500 × g for 15 min. Supernatant plasma was stored at −80°C before analysis.

In total, 38 subjects (AKI group = 17; reference (REF) group = 21) were used for the initial screen. Among them, 17 subjects of the AKI group came from the discovery cohort, and 21 subjects of the control group were randomly selected from 114 patients without CSA-AKI in the discovery cohort. In the discovery cohort, a total of 6 plasma biomarkers [such as, growth differentiation factor 15 (GDF15), soluble ST2 (interleukin 1 receptor-like 1) (sST2), soluble urokinase plasminogen activator receptor (uPAR), lipocalin 2 (NGAL), fatty acid binding protein 3 (FABP3), and interleukin 6 (IL6)] were measured using the Quantibody Custom Array (RayBio; RayBiotech, GA, USA) according to the instructions of manufacturer. Promising biomarkers in the discovery cohort were further validated in the validation cohort (AKI = 50; REF = 38) using ELISA assays, where 50 patients of the AKI group came from the validation cohort and 38 patients of the control group were randomly selected from 244 patients without AKI after cardiac surgery in the validation cohort.

### Enzyme-Linked Immunosorbent Assay (ELISA) Detection

ELISA kits for the measurement of GDF-15 (70-EK1100-96), IL1RL1 (RK-KOA0636), and uPAR (70-EK1171-96) were purchased from Multi Sciences (Lianke) Biotech Ltd. (Hangzhou, China). ELISAs were performed according to guidelines provided by the manufacturer.

### RNA Extraction, cDNA Synthesis, and Real-Time Quantitative PCR (RT-qPCR)

The density gradient centrifugation by Ficoll-Hypaque (Sigma Chemical Co, St Louis, MO, USA) was applied for peripheral blood mononuclear cells (PBMCs) separation from the blood of AKI and control subjects. RNA was isolated from PBMCs obtained from the patients using Trizol (Invitrogen, Carlsbad, CA, USA) and reverse transcribed using the RevertAid First Strand cDNA Synthesis Kit (Thermo Fisher Scientific, Waltham, MA, USA). The RT-qPCR analysis was performed using AceQ Universal SYBR qPCR Master Mix (Vazyme, Nanjing, China), primers, and the C1000 Touch™ thermal cycler (Bio-Rad, Hercules, CA, USA). The data were normalized to GAPDH levels within each sample and analyzed using the ΔΔCt method. Primer sequences are listed as follows: GDF-15: forward 5′-GACCCTCAGAGTTGCACTCC-3′, reverse 5′-GCCTGGTTAGCAGGTCCTC-3′; IL1RL1: forward 5′-GAAAACCTAGTTACACCGTGGAT-3′, reverse 5′-GCAAACACACGATTTCTTTCCTG-3′; uPAR: forward 5′-GAGCTATCGGACTGGCTTGAA-3′, reverse 5′-CGGCTTCGGGAATAGGTGAC-3′.

### Statistical Analysis

For continuous variables, normally distributed variables are summarized as the mean and SD, and non-normally distributed variables are shown as the median and interquartile range (IQR). Categorical variables are expressed as frequencies with proportions. The unpaired Student's *t*-test and Mann–Whitney *U*-test were applied to compare the difference between the two groups. Three-group comparisons were accomplished by analysis of variance or the Kruskal–Wallis test. The Fisher's exact test or chi-square test was adopted to evaluate the association between two categorical variables. Simple logistic regression was used to examine the association between each biomarker and AKI. Multivariate logistic regression models, such as biomarkers and clinical factors were constructed to achieve better performance of prediction. We conducted leave-one-out cross-validation (LOOCV) with logistic regression. The goodness-of-fit of the models was assessed by the Akaike information criterion (AIC) and the Bayesian information criterion (BIC). A better model ought to have lower AIC and BIC statistics. Receiver operating characteristic (ROC) curve analysis was conducted to evaluate the model discrimination. The area under the curve (AUC), which is equal to the C-statistic, was calculated. The Brier score was used to assess the calibration of the model. In addition, ROC curves were formulated to assess the discriminability of each selected biomarker for predicting AKI. Based on the Youden index, the optimal cutoff values were determined. Afterward, according to these cut-off values, the specificity, and sensitivity of each biomarker for predicting AKI. Spearman's test was performed to examine the correlation between the selected biomarkers and AKI (defined as creatinine alteration). Multivariable logistic regression was used to examine whether the association between each biomarker and AKI was independent of other clinical cofounding factors. The odds ratios (*OR*s) with 95% *CI*s of each biomarker are reported. The data were processed using statistical packages in R (version 4.0.2), and diagrams were plotted by ggplot2 package in R. Significance levels were set at *p* < 0.05 for 2-tailed tests in all analyses.

## Results

### WGCNA and DEG Analysis

**Figure 2:**
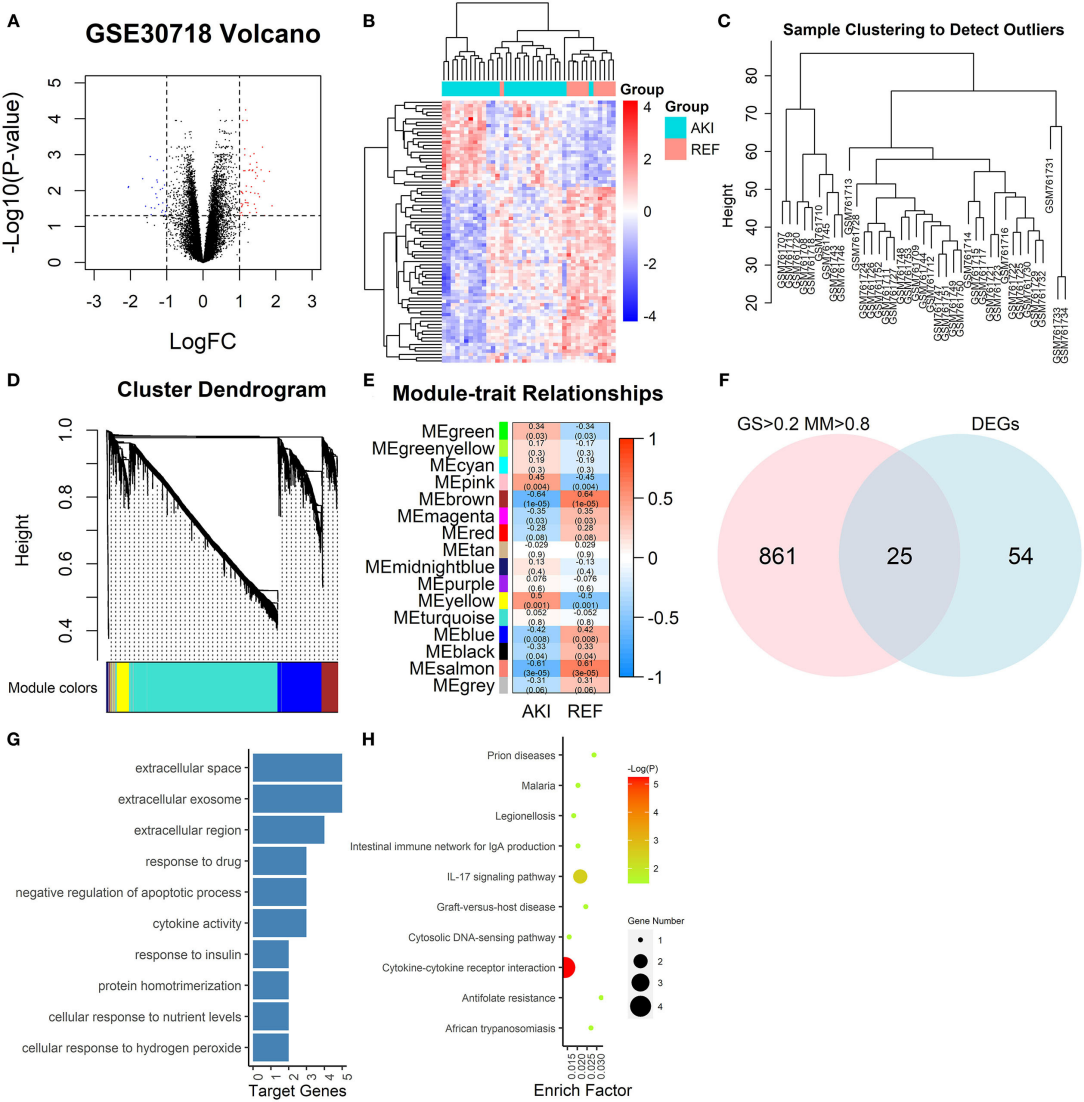
Graphs depicting bioinformatics analysis. **(A)** The volcano plots. The red dots and blue dots represent differentially expressed genes (DEGs) (|logFC| > 1, adj.*p* < 0.05). **(B)** Heatmap of all DEGs. **(C)** A clustering diagram of samples to detect outliers of GSE30718 in weighted gene co-expression network analysis (WGCNA) analysis. **(D)** Clustering dendrogram of genes based on topological overlapping. A total of 15 modules were identified. **(E)** A heatmap of the correlation between module features and clinical information of acute kidney injury (AKI). Each row corresponds to a module eigengene, and each column corresponds to a trait. Each cell contains the corresponding correlation and *p*. The table is color-coded by correlation according to the color legend. **(F)** Venn diagram of DEGs and GS > 0.2, MM > 0.8. **(G)** GO analysis of key module genes. The bar plot indicates the number of enriched genes in the significant pathways (*p* < 0.05). The *x*-axis shows the number of genes enriched in this term. **(H)** Kyoto Encyclopedia of Genes and Genomes (KEGG) pathway enrichment analysis of key modules. The node size reflects the gene count, and the node color reflects the *p* [–log10 (*p*)]. DEGs, differentially expressed genes; GO, gene ontology; KEGG, kyoto encyclopedia of genes and genomes; GS, gene significance; MM, module membership. WGCNA, weighted gene co-expression network analysis.

The GEO dataset GSE30718 (17) included 26 patients with AKI and 11 control patients. Then, 79 DEGs between the AKI group and control group were extracted (|logFC| > 1, adj.*p* < 0.05), among which 52 genes were upregulated and 27 genes were downregulated (Figures 2A,B). The 20,188 genes in GSE30718 were screened by median absolute deviation (MAD) to reduce noise and the top 10,094 highly genes were extracted for further analysis. We calculated the Pearson's correlation coefficient to cluster the samples in GSE30718. We drew a sample clustering tree and found no outliers (Figure 2C). We set the soft threshold to 8 and constructed a scale-free network. Finally, 18 modules were identified with average hierarchical clustering and dynamic tree clipping. Five modules showed significant correlations with AKI (*p* < 0.01), such as salmon, blue, yellow, brown, and pink modules (Figures 2D,E). The 25 hub genes are identified by the criterion of AKI based GS > 0.2 and MM > 0.8 with a threshold of *p* < 0.05 in 5 key modules (Figures 2F-H).

### Identification and Dataset Validation of Candidate Genes

We got GDF15 from hub genes, which was previously reported to be related to AKI, (18, 19) and designated IL1RL1, uPAR, FABP3, IL6, and NGAL from other articles (9, 20–22).

### Clinical Characteristics of the Study Cohorts

The clinical characteristics of patients from the two cohorts are summarized in Table 1. The clinical characteristics of the two groups are comparable. No significant differences were observed among the control and AKI groups in age, sex, cardiac function, left ventricular ejection fraction, diabetes, hypertension, previous myocardial infarction, and CPB time. In addition, the AKI group had significantly prolonged hospital duration, ventilation time, and ICU stay.

**Table 1:** Clinical characteristics of the patients.

	**Validation cohort**			**Discovery cohort**		
	**AKI (***N*** = 50)**	**Non-AKI (***N*** = 38)**	***P***	**AKI (***N*** = 17)**	**REF (***N*** = 21)**	***P***
Age, y, median (IQR)	65 (58–72)	61 (56–67)	0.198	64 (59–69)	60 (56–66)	0.483
Male, *n* (%)	32 (64.0%)	23 (60.5%)	1	13.0 (76.5%)	13.0 (61.9%)	0.542
LVEF, %, median (IQR)	61 (55–68)	64 (59–68)	0.353	57 (50–61)	62 (56–68)	0.202
NYHA class III or IV, *n* (%)	16 (32.0%)	6 (15.8%)	0.127	7.00 (41.2%)	3.00 (14.3%)	0.133
Diabetes mellitus, *n* (%)	4 (8.0%)	1 (2.6%)	0.559	0 (0%)	0 (0%)	1
Hypertension, *n* (%)	20 (40.0%)	10 (26.3%)	0.303	8.00 (47.1%)	4.00 (19.0%)	0.135
Previous heart surgery, *n* (%)	9 (18.0%)	2 (5.3%)	0.155	3.00 (17.6%)	0 (0%)	0.161
CPB time, min, median (IQR)	120 (94–140)	110 (84–130)	0.127	120 (78–140)	110 (83–120)	0.246
CPB time > 120 min, *n* (%)	21 (42.0%)	14 (36.8%)	0.098	8.00 (47.1%)	6.00 (28.6%)	0.22
RRT or in-hospital death, *n* (%)	1 (2.0%)	0 (0%)	1	1.00 (5.9%)	0 (0%)	0.915
Hospitalized time, d, median (IQR)	18 (14–21)	14 (13–18)	0.009	17 (11–20)	14 (13–18)	0.27
ICU stay, d, median (IQR)	4 (3.0–5.0)	3 (2.0–3.0)	0.006	3.0 (2.0–4.0)	2.0 (2.0–3.0)	0.145
ventilation time, h, median (IQR)	21 (16–27)	18 (13–21)	0.008	21 (12–24)	19 (15–21)	0.113

*AKI, acute kidney injury; REF, reference; CPB, cardiopulmonary bypass; IQR, interquartile range; RRT, renal replacement therapy; LVEF, left ventricular ejection fractions; CABG, coronary artery bypass graft; ICU, intensive care unit; NYHA, new york heart association*.

### Potential Blood Biomarker Markers for AKI

**Figure 3:**
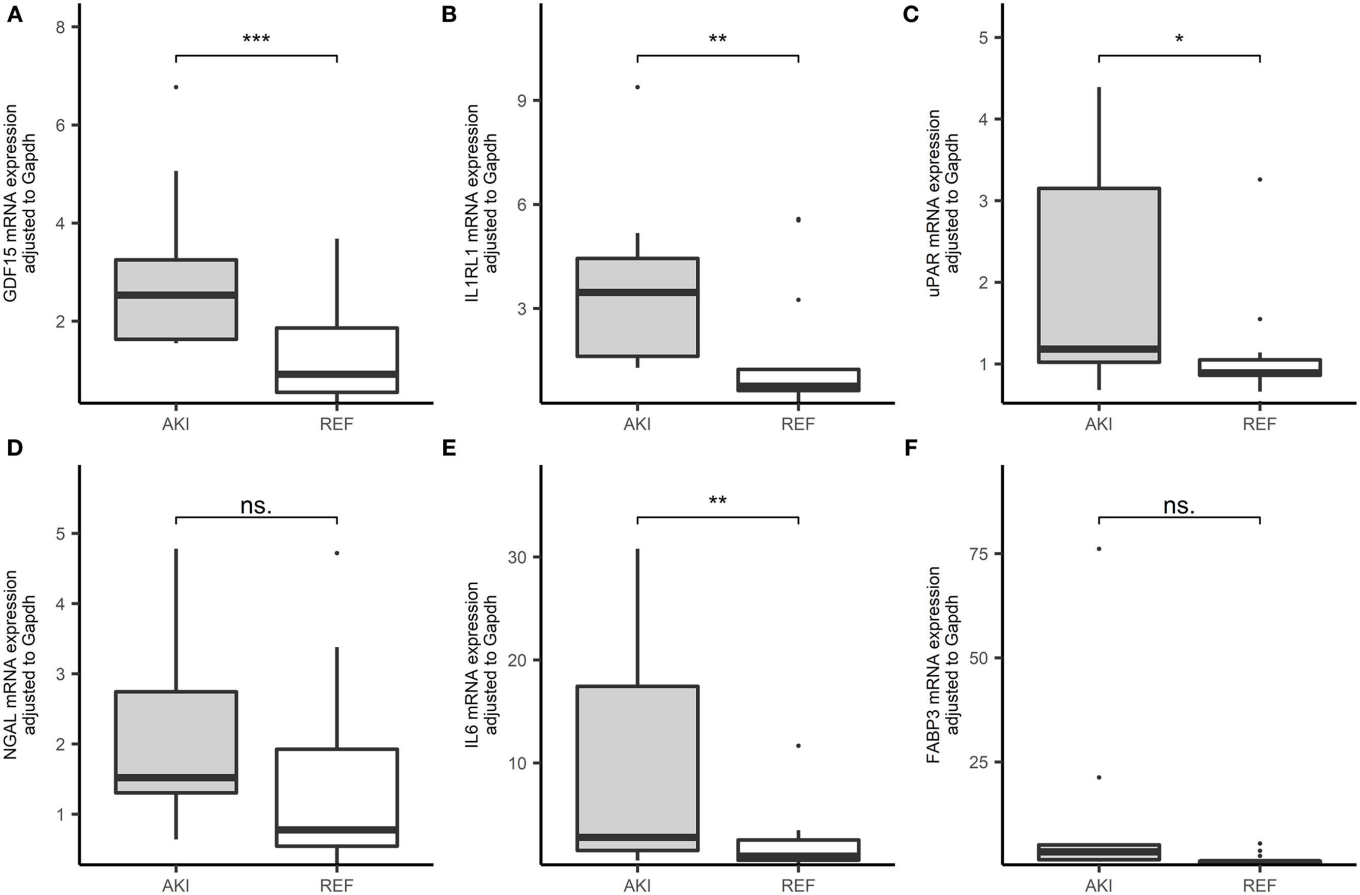
Boxplots of the RNA expression level of 6 candidate genes in our cohorts (AKI = 19, REF = 27). **(A)** GDF-15 expressional level in peripheral blood in patients after cardiac surgery in the cohort. **(B)** IL1RL1 expressional level in peripheral blood in patients after cardiac surgery in the cohort. **(C)** The urokinase plasminogen activator receptor (uPAR) expressional level in peripheral blood in patients after cardiac surgery in the cohort. **(D)** Neutrophil gelatinase-associated lipocalin (NGAL) expressional level in peripheral blood in patients after cardiac surgery in the cohort. **(E)** Interleukin 6 (IL6) expressional level in peripheral blood in patients after cardiac surgery in the cohort. **(F)** Fatty acid binding protein 3 (FABP3) expressional level in peripheral blood in patients after cardiac surgery in the cohort. ***p < 0.001, **p < 0.01, *p < 0.05, ns. *p* > 0.05.

We inspected 6 candidate biomarkers' expression levels in PBMCs within the cohort, such as GDF-15, IL1RL1, uPAR, NGAL, IL6, and FABP3 and patients who developed AKI had significantly higher GDF-15, IL1RL1, uPAR, and IL6 expression in comparison with the patients without AKI (Figure 3).

**Figure 4:**
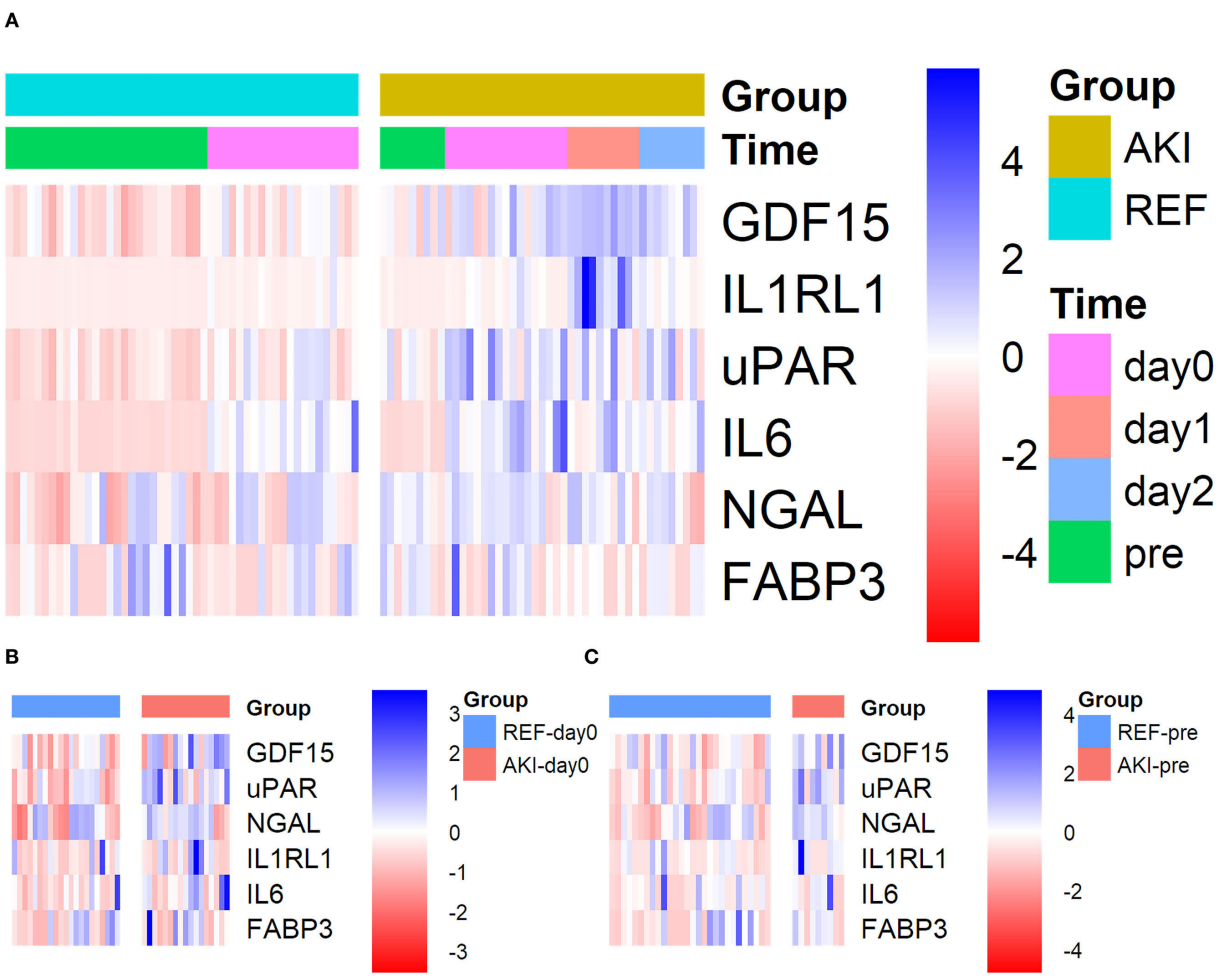
Heatmap showing the plasma levels of six candidate biomarkers in the discovery cohort using Quantibody Custom Array. Plasma levels of the AKI group (*n* = 17) and control group (*n* = 21) at different time points were measured. Blue color indicates lower and red color indicates higher protein expression, with intensities signifying magnitude of the change. **(A)** All 4 time points, **(B)** pre (pre-operative), and **(C)** day0 [at admission to intensive care unit (ICU)]. AKI, acute kidney injury.

**Figure 5:**
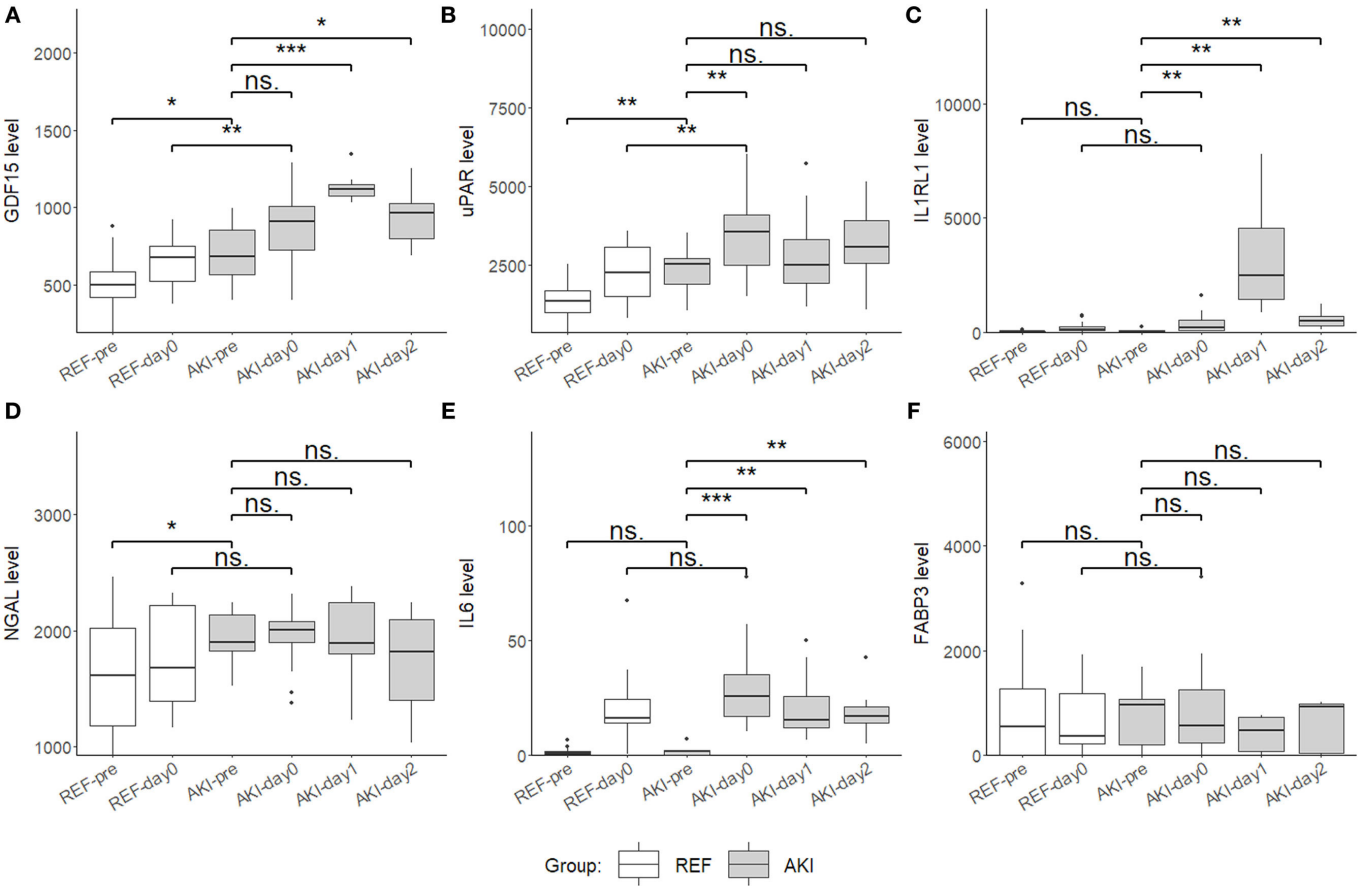
Boxplots showing the plasma level of six candidate biomarkers in the discovery cohort using Quantibody Custom Array. **(A)** Growth differentiation factor 15 (GDF15), **(B)** uPAR, **(C)** IL1RL1, **(D)** NGAL, **(E)** IL6, and **(F)** FABP3 plasma levels of AKI group (*n* = 17) and control group (*n* = 21) at 4 time point, pre (pre-operation), day0 (ICU arrival after surgery), day1 and day2, were measured and compared. Boxplot represents the median and interquartile range (IQR), whiskers adjacent values (extreme values within 1.5 IQR of nearest quartile), and points outside values. ***p < 0.001, **p < 0.01, *p < 0.05, ns. *p* > 0.05.

**Figure 6:**
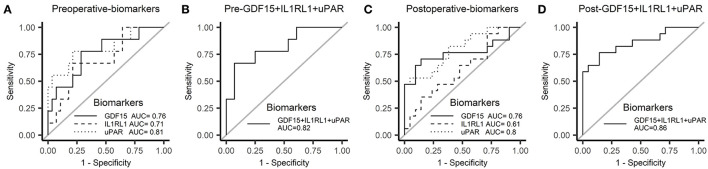
Receiver operator characteristic (ROC) curve for AKI in the discovery cohort. **(A)** ROC curve of pre-operative biomarkers for AKI. **(B)** ROC curve of pre-operative biomarkers combination for AKI. **(C)** ROC curve of post-operative biomarkers for AKI. **(D)** ROC curve of post-operative biomarkers combination for AKI. ROC, receiver operator characteristic.

For further validation in the plasma, these six candidate biomarkers at different time points were measured in the discovery cohort using Quantibody Custom Array (Figure 4). The post-operative concentrations of GDF-15 and uPAR were found significantly elevated in patients with AKI. Additionally, pre-operative GDF-15, IL1RL1, and NGAL were significantly higher in the AKI group (Figure 5). Moreover, these biomarkers were shown to have prediction ability for AKI (AUC > 0.6) and their combination has a bigger AUC (pre-biomarkers: AUC: 0.82; post-biomarkers: AUC: 0.86) (Figure 6).

### Performance of Candidate Biomarkers in Tracking AKI in Patients After Cardiac Surgery

**Figure 7:**
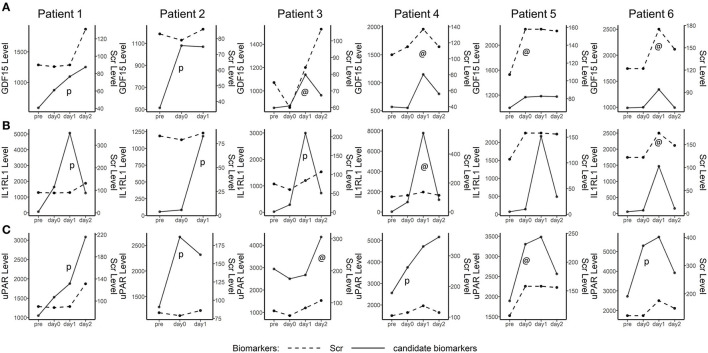
Line plot depicting plasma GDF15, IL1RL1, and uPAR in five detected AKI after cardiac surgery patients. The time point is shown on the *x*-axis, while the biomarker levels and serum creatinine (SCr) levels are indicated on the left and right vertical axes, respectively. The perioperative tracking plots demonstrated the fluctuations of plasma GDF15, IL1RL1, and uPAR along with SCr and NGAL over time. @, candidate biomarkers increased simultaneously with the increase of SCr. *p*, increasing of candidate biomarkers preceded Scr.

To interrogate the early diagnosis value of these 3 candidate biomarkers, the tracking plots (Figure 7) show that the perioperative fluctuations of these candidate biomarkers along with SCr. In some cases, these proteins increase simultaneously (marked by “@”) with the elevation of SCr and in many other cases, these proteins preceded the SCr (marked by “p”). The three plasma biomarkers measured did well, preceding or coinciding with an elevation of SCr in most patients. In some cases, the fluctuations of plasma biomarkers were substantially more noticeable than the subtle changes in SCr.

### Validation of the Biomarker-Based Predictive Model for AKI

**Figure 8:**
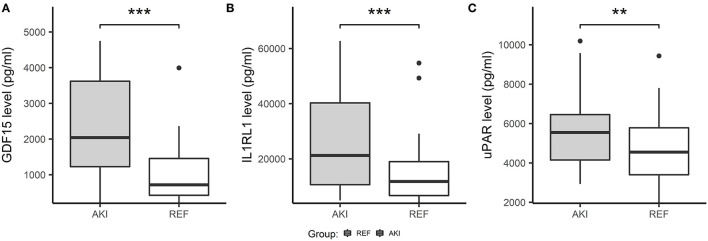
Boxplots showing plasma concentration of three candidate biomarkers in validation cohorts using ELISA. Three candidate biomarkers were measured and compared in the AKI group (*n* = 50) and control group (*n* = 38) at admission to ICU. **(A)** The concentration of GDF15 was significantly increased in the AKI group compared with the non-AKI group. **(B)** Concentration of IL1RL1 was significantly increased in the AKI group compared with the non-AKI group. **(C)** Concentration of uPAR were significantly increased in the AKI group compared with the non-AKI group. ***p < 0.001, **p < 0.01, *p < 0.05, ns. *p* > 0.05.

**Figure 9:**
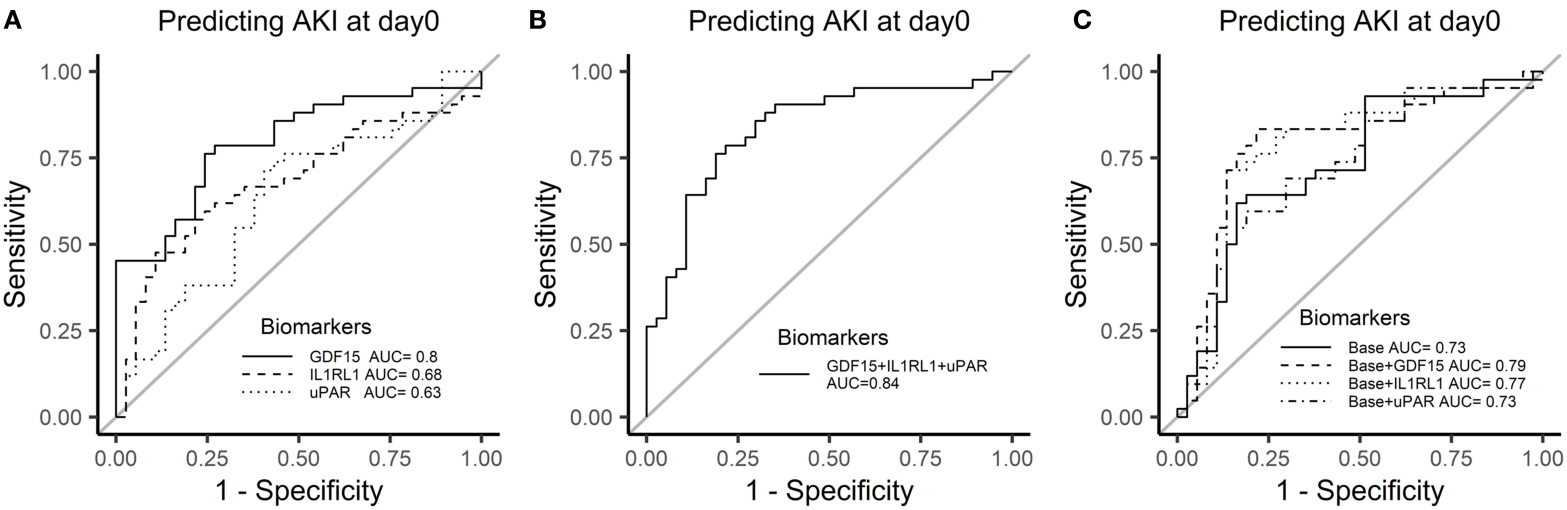
Receiver operator characteristic curves of 3 candidate biomarkers at admission to ICU for AKI in the validation cohort. **(A)** ROC curve of biomarkers for AKI. **(B)** ROC curve of biomarkers combination for AKI. **(C)** Incremental changes to the ROC curve for AKI by adding plasma biomarkers to the traditional clinical model in the discovery cohort.

We further validated these 3 potential biomarkers GDF-15, IL1RL1, and uPAR in the patients from the validation cohort. Patients with CSA-AKI had significantly higher concentrations of GDF-15, IL1RL1, and uPAR than patients without AKI (Figure 8). The area under the ROC curves, represented by the C-statistic, indicated that at admission to ICU, these biomarkers had good discriminability for CSA-AKI, with GDF-15 (C-statistic: 0.8, 95% *CI*, 0.7–0.89), IL1RL1 (C-statistic: 0.68; 95% *CI*, 0.56–0.8), uPAR (C-statistic: 0.63; 95% *CI*, 0.5–0.76) and their combination (C-statistic: 0.84; 95% *CI*, 0.74–0.93) (Figures 9A,B).

The previous studies of the same center have constructed a clinical model based on the traditional clinical factors from Cleveland Clinic (11), the Metha (12), and the Birnie scores (13) to predict AKI after cardiac surgery, such as age, sex, left ventricular ejection fraction, diabetes, hypertension, previous cardiac surgery, estimated GFR (eGFR), and hemoglobin (23). We constructed a similar clinical-factor-based model with similar prediction ability for AKI (C-statistic: 0.73, 95% *CI*, 0.62–0.85). The combination of GDF-15 or IL1RL1 with clinical parameters had a better power to discriminate between the two groups than the model only with clinical parameters (base: 0.73; base + GDF-15: 0.79; base + IL1RL1: 0.77) (Figure 9C).

At last, 3 multivariate logistic regression models, such as the biomarker-based model (Model 1), clinical-factor-based model (Model 2), and the mixed model (Model 3) were developed and compared. Compared with Model 2, Model 1 yielded lower AIC and BIC values and had better calibration and discrimination performance [C-statistic; Model 1, 0.84 (0.74–0.93); Model 2, 0.73 (0.62–0.85)]. Moreover, the mixed model (Model 3) performed better than Model 1 and Model 2 (Table 2).

**Table 2:** Predicting AKI at admission to ICU in the validation cohort.

	**C-statistics (95%CI)**	**Sensitivity**	**Specificity**	**Accuracy**	**AIC**	**BIC**	**Brier**
GDF15	0.8 (0.7–0.89)	0.76	0.76	0.78	83	88	0.17
IL1RL1	0.68 (0.56–0.8)	0.62	0.73	0.72	99	104	0.21
uPAR	0.63 (0.5–0.76)	0.71	0.59	0.67	106	110	0.23
Model 1	0.84 (0.74–0.93)	0.76	0.81	0.82	81	90	0.15
Model 2	0.73 (0.62–0.85)	0.64	0.81	0.79	98	124	0.16
Model 3	0.86 (0.77–0.94)	0.83	0.84	0.85	80	99	0.12

*AIC, akaike information criterion; BIC, bayesian information criterion; ICU, intensive care unit. Model 1: GDF15, IL1RL1, and uPAR; Model 2: age, sex, hypertension, diabetes, estimated glomerular filtration rate (eGFR), hemoglobin, and previous heart disease; Model 3: GDF15, IL1RL1, uPAR, eGFR, and hemoglobin*.

Additionally, these biomarkers were investigated in the risk stratification of AKI. In multivariate logistic regression analyses, after adjusting for cofounders, the third and fourth quantiles of GDF-15 showed an 8.01-fold risk and a 161.36-fold risk of AKI, respectively. The third and fourth quantiles of IL1RL1 held a 5.8-fold risk and a 25.56-fold risk of AKI compared with the first quantile. The third and fourth quantiles of IL1RL1 held a 5.8-fold risk and a 25.56-fold risk of AKI compared with the first quantile. The fourth quantile of uPAR held a 6.87-fold risk of AKI compared with the first quantile (Table 3).

**Table 3:** Multivariate logistic regression analyses of selected biomarkers for predicting higher risk of AKI.

	**Range, pg/ml**	**OR (95% CI)**	***p***	**Adjusted^a^ OR (95% CI)**	**Adjusted** ***p***
GDF15	Continuous	5.59 (3–13.9)	0	6.64 (3–19.4)	0.0001
Q1	(203, 522)	1	Ref	1	Ref
Q2	(522, 1.37e+03)	4.36 (1.04–23.21)	0.0567	5.82 (1.19–37.35)	0.0401
Q3	(1.37e+03, 2.28e+03)	7.33 (1.73–39.9)	0.0109	8.01 (1.6–53.17)	0.0171
Q4	(2.28e+03, 4.75e+03)	101.33 (13.76–2231)	1e−04	161.36 (16.89–4295.26)	1e−04
IL1RL1	Continuous	2.86 (2–6.07)	0.0018	3.59 (2–8.4)	0.0007
Q1	(3.93e+03, 9.19e+03)	1	Ref	1	Ref
Q2	(9.19e+03, 1.63e+04)	1.44 (0.39–5.59)	0.5844	2.21 (0.48–11.42)	0.3169
Q3	(1.63e+04, 2.73e+04)	2.98 (0.81–11.88)	0.1073	5.8 (1.25–32.5)	0.032
Q4	(2.73e+04, 6.27e+04)	12.28 (2.85–69.26)	0.0017	25.56 (4.86–185.8)	4e−04
UPAR	Continuous	1.96 (1–3.46)	0.0115	2.15 (1–4.21)	0.0146
Q1	(1.95e+03, 3.69e+03)	1	Ref	1	Ref
Q2	(3.69e+03, 5.26e+03)	1.4 (0.39–5.19)	0.6051	2.22 (0.48–11.53)	0.3184
Q3	(5.26e+03, 6.26e+03)	2.36 (0.65–9.05)	0.1972	4.61 (1.01–24.84)	0.0581
Q4	(6.26e+03, 1.02e+04)	5.14 (1.36–22.07)	0.0197	6.87 (1.57–36.43)	0.015

*OR, odds ratio; Q1, the first quartile; Q2, the second quartile; Q3, the third quartile; Q4, the fourth quartile; RRT, renal replacement therapy; GDF-15, growth/differentiation factor-15; IL1RL1, interleukin 1 receptor like 1; UPAR, urokinase plasminogen activator*.

a *Adjusted for age, sex, diabetes, hypertension, previous heart disease, eGFR, and hemoglobin*.

## Discussion

Weighted Gene Co-expression Network Analysis was employed in the current study to mine the hub gene. Together with 5 potential biomarkers from previous articles, we evaluated these 6 candidates by multiplex cytokine array and ELISA. Then, we developed and validated a novel predictive model incorporating uPAR, GDF15, and IL1RL1 at admission to ICU for predicting CSA-AKI, which outperformed the traditional clinical-factor-based model. This study offers an early predictive method for CSA-AKI and shows that uPAR, GDF15, and IL1RL1 were able to predict AKI and higher concentrations of them were associated with higher risks of AKI after cardiac surgery.

Cardiac-associated acute kidney injury, a threatening post-operative complication, is independently associated with prolonged mechanical ventilation, increased days of intensive care, and increased post-operative mortality. Evidence is supporting that those who get only transient post-operative AKI at stage 1 or 2 take a significantly increased risk of death and a higher risk of chronic kidney disease (4, 5). Due to the delayed CSA-AKI diagnosis, many efforts have been made to construct novel biomarkers model for CSA-AKI prediction. In this study, GDF-15 was screened out with WGCNA analysis. GDF-15 (MIC-1), a cytokine expressed in various tissues, is upregulated upon stimuli, such as myocardial stretch, volume overload, ischemia/reperfusion, and oxidative stress (18). Former research showed that pre-operative plasma GDF-15 predicted CSA-AKI independently (19). Its part in an early renoprotective injury response of AKI indicates post-operative GDF-15's potential for early detection of AKI (18). sST2, the soluble form of ST2 (also known as IL1RL1), has an important part in the regulation of immune and inflammatory response. Pre-operative sST2 was validated to stratify patients at higher risk of getting CSA-AKI (20). Soluble uPAR, produced by the cleavage of the membrane-bound uPAR, is found in different body fluids, such as blood and urine. As a potential biomarker of immune activation and inflammation in multiple disease states, pre-operative suPAR recently was identified as an independent risk biomarker predicting incident kidney diseases in the emergency department and was associated with the development and severity of CSA-AKI (21, 22).

Nephrocheck (Astute Medical, San Diego, CA, USA), containing tissue inhibitor of metalloproteinases 2 (TIMP-2) and insulin-like growth factor binding protein 7 (IGFBP7), has been accepted as the first diagnostic test for AKI approved by the US Food and Drug Administration (24). In a recent meta-analysis for the diagnosis of CSA-AKI in adults by urinary TIMP-2 and IGFBP7 (25), when outcomes are restricted to KDIGO stage 1 criteria similar to our study, five studies provided a relatively low estimated AUC (AUC: 0.835; sensitivity: 0.73; and specificity: 0.76), which is comparable with our biomarker-based model (AUC: 0.84; sensitivity: 0.76; and specificity: 0.81). Many other biomarkers were reported to have diagnostic value for CSA-AKI in recent years (9). In a high-quality meta-analysis (26), plasma and urine NGAL (AUC: 0.71; 95% *CI*, 0.64–0.77 and AUC:0.72; 95% *CI*, 0.66–0.79, respectively), plasma and urine plasma cystatin C (AUC: 0.69; 95% *CI*, 0.63–0.74 and AUC: 0.63; 95% *CI*, 0.37–0.89, respectively), urine KIM-1 (AUC: 0.72; 95% *CI*, 0.59–0.84), and urine L-FABP (AUC: 0.72; 95% *CI*, 0.60–0.85) showed moderate diagnostic value for CSA-AKI. In our study, plasma GDF-15 performed better than these biomarkers (AUC: 0.8, 95% *CI*, 0.7–0.89; sensitivity: 0.76; and specificity: 0.76).

When it came to the measurement of time points, the frequently used time points of collecting samples for the prediction of CSA-AKI were at admission to ICU, 4 h and 12 h after cardiac surgery. Among these [TIMP-2^*^IGFBP7] studies for the diagnosis of CSA-AKI in adults, only one study (27) showed prediction value of TIMP-2 and IGFBP7 at admission to ICU (AUC: 0.74; sensitivity: 0.60; and specificity: 0.88) and other positive outcomes occurred at 4 h after cardiac surgery or later (25, 27–30). Additionally, there were contradictory results at 4 h after cardiac surgery where no significant differences of [TIMP-2^*^IGFBP7] were found between the AKI group and those without AKI (31, 32). Moreover, in our study, the plasma level of NGAL did not upregulate significantly in patients with CSA-AKI as previous articles showed, which happened several times before in the early post-operative period plasma of adults undergoing cardiac surgery (33, 34). These indicated that urine [TIMP-2^*^IGFBP7] and plasma NGAL may not be a good early prediction factor for CSA-AKI in adults while our biomarker-based model showed potential to predict AKI at admission to ICU. In our study, biomarkers at 4 time points were measured in the discovery cohort, and biomarker at admission to ICU showed the perioperative tracking plots were made to show part of patients with AKI. In these cases, candidate biomarkers either increased simultaneously with the increase of SCr or preceded the SCr (Figure 7).

Admittedly, our research had some limitations. First, we accepted patients from a single center and external validation from other hospitals should be further performed. Second, the number of patients in our study is small. The performance of the biomarker-based model for early diagnosis of CSA-AKI requires an evaluation in a larger multicenter and prospective study. Third, in our study, AKI was defined according to the KDIGO guideline while the diagnosis of AKI is recently challenged by the fact that creatinine is not the optimal biomarker for renal function impairment. Therefore, there remains the possibility that patients in the non-AKI group have AKI in our study.

## Conclusions

In this research, we developed and validated a multi-biomarker predictive model containing GDF-15, IL1RL1, and uPAR for predicting CSA-AKI, which outperformed the traditional clinical-factor-based model.

## Data Availability Statement

Publicly available datasets were analyzed in this study. This data can be found here: https://www.ncbi.nlm.nih.gov/geo/query/acc.cgi?acc=GSE30718.

## Ethics Statement

The studies involving human participants were reviewed and approved by the Research Ethics Committee of the First Affiliated Hospital, College of Medicine, Zhejiang University (No. 20211318). The patients/participants provided their written informed consent to participate in this study.

## Author Contributions

HZ and QS designed the study. CW, HC, LS, LM, TZ, and WC were responsible for the experiments. YZ performed the statistical analysis and wrote the manuscript, which was revised by every author iteratively. The research was supervised by HJ and JC. All authors approved the final manuscript.

## Funding

This study was supported by the key research project of precision medicine in the National Key Research and Development Plan (2017YFC0907603), the Natural Science Foundation of China (81900611), LQ19H050008, and 2018M642464, and Zhejiang Provincial Natural Science Foundation of China under Grant, LQ19H050004.

## Conflict of Interest

The authors declare that the research was conducted in the absence of any commercial or financial relationships that could be construed as a potential conflict of interest.

## Publisher's Note

All claims expressed in this article are solely those of the authors and do not necessarily represent those of their affiliated organizations, or those of the publisher, the editors and the reviewers. Any product that may be evaluated in this article, or claim that may be made by its manufacturer, is not guaranteed or endorsed by the publisher.

## Abbreviations

AKI: acute kidney injury
ICU: intensive care unit
GDF15: growth differentiation factor 15
IL1RL1: interleukin 1 receptor-like 1
uPAR: soluble urokinase plasminogen activator receptor
KDIGO: Kidney Disease: Improving Global Outcomes (KDIGO) Guidelines
NGAL: neutrophil gelatinase-associated lipocalin
FABP3: fatty acid binding protein 3
IL6: interleukin 6
AIC: Akaike information criterion
BIC: Bayesian information criterion
ROC: receiver operating characteristic curve
*OR*: odds ratio
WGCNA: Weighted gene co-expression network analysis
CPB: cardiopulmonary bypass.
